# A detailed insight of the tumor targeting using nanocarrier drug delivery system

**DOI:** 10.1080/10717544.2023.2183815

**Published:** 2023-03-03

**Authors:** Sibgha Batool, Saba Sohail, Fakhar ud Din, Ali H. Alamri, Ahmad S. Alqahtani, Mohammad A. Alshahrani, Mohammed A. Alshehri, Han Gon Choi

**Affiliations:** a Department of Pharmacy, Faculty of Biological Sciences, Quaid-i-Azam University, Islamabad, Pakistan; b Nanomedicine Research Group, Department of Pharmacy, Faculty of Biological Sciences, Quaid-i-Azam University, Islamabad, Pakistan; c Department of Pharmaceutics, College of Pharmacy, King Khalid University, Abha, Saudi Arabia; d Department of Pharmacy, Mental Health Hospital, Ministry of Health, Abha, Saudi Arabia; e Department of Medical Supply in Khamis Mushet General Hospital, Ministry of Health, Khamis Mushet, Saudi Arabia; f Department of Pharmacy, Abha Maternity and Children Hospital, Ministry of Health, Abha, Saudi Arabia; g College of Pharmacy & Institute of Pharmaceutical Science and Technology, Hanyang University, Ansan, South Korea

**Keywords:** Tumor, nanotechnology, targeting, drug delivery, pharmaceutics

## Abstract

Human struggle against the deadly disease conditions is continued since ages. The contribution of science and technology in fighting against these diseases cannot be ignored exclusively due to the invention of novel procedure and products, extending their size ranges from micro to nano. Recently nanotechnology has been gaining more consideration for its ability to diagnose and treat different cancers. Different nanoparticles have been used to evade the issues related with conservative anticancer delivery systems, including their nonspecificity, adverse effects and burst release. These nanocarriers including, solid lipid nanoparticles (SLNs), liposomes, nano lipid carriers (NLCs), nano micelles, nanocomposites, polymeric and magnetic nanocarriers, have brought revolutions in antitumor drug delivery. Nanocarriers improved the therapeutic efficacy of anticancer drugs with better accumulation at the specific site with sustained release, improved bioavailability and apoptosis of the cancer cells while bypassing the normal cells. In this review, the cancer targeting techniques and surface modification on nanoparticles are discussed briefly with possible challenges and opportunities. It can be concluded that understanding the role of nanomedicine in tumor treatment is significant, and therefore, the modern progressions in this arena is essential to be considered for a prosperous today and an affluent future of tumor patients.

## 1. Introduction

**Figure 1.**
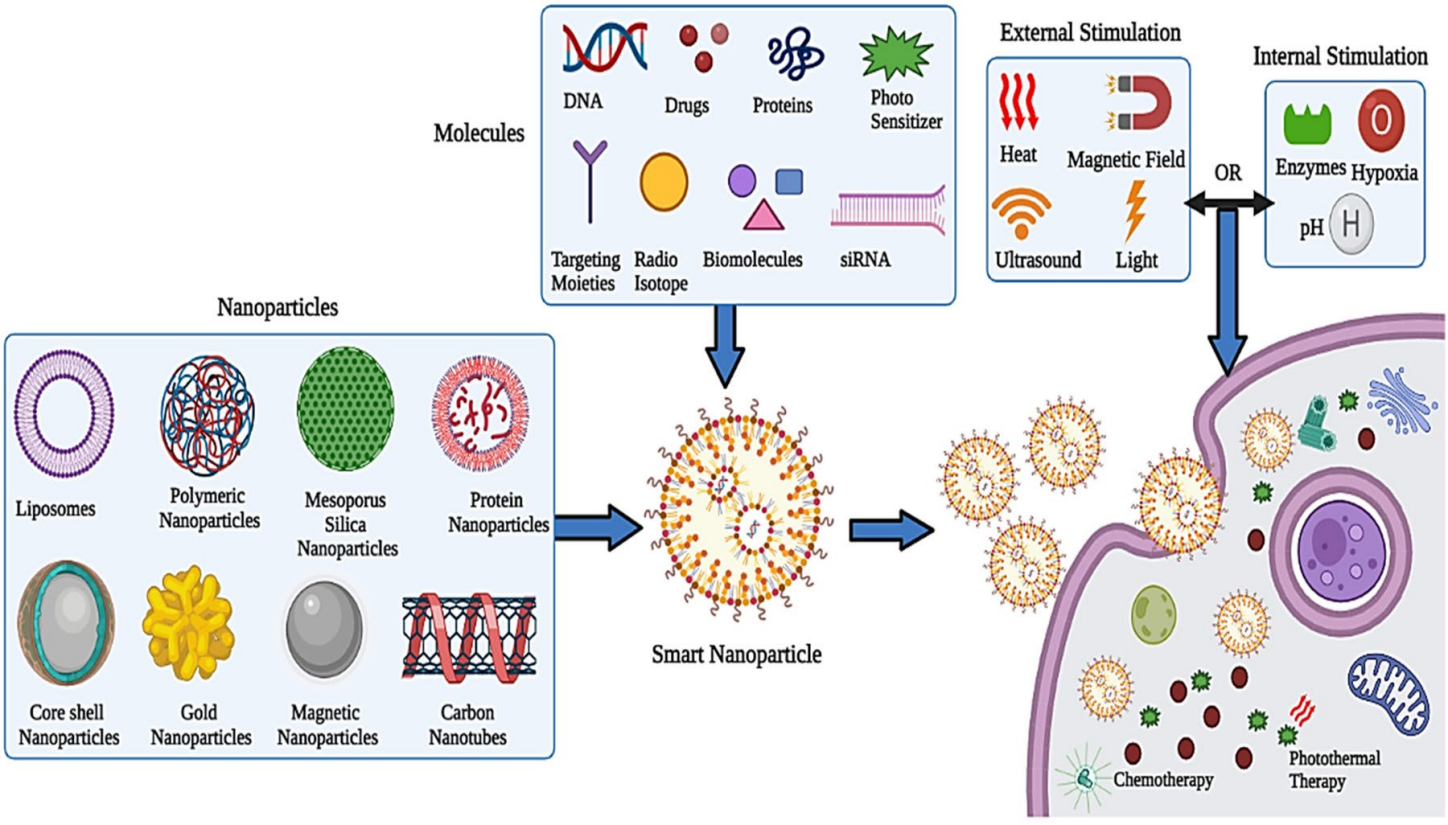
Graphical representation of the nanomedicine targeting tumor (Created with BioRender).

Cancer is an incurable disease owing to its endless features that may be activated by both factors (endogenous and exogenous). It is likely to exceed cardiac problems, which are now thought to be the main reason of death globally, as it is the second foremost reason of fatality, accounting for almost 9.6 million deaths in 2018 (Bhakta et al., 2015). Lung cancer, which accounts for around 1.76 million cancer-related deaths worldwide, is accompanied by colorectal tumor 860,000, stomach tumor 784,000, liver tumor 781,000 and breast tumor 628,000 deaths respectively. About 70% of deaths in different countries are endorsed to cancer, by 2030, there may be 21 million cancer sufferers worldwide (Jazieh et al., 2019). The high cost of treating cancer patients as well as the palliative care issues result in a significant financial burden. In 2015, it was predicted that cancer has an annual economic cost of almost US$100 billion (Jazieh et al., 2019). Thus these require the investigation of safer, newer and new effective diagnostic and therapeutic methods for fighting the disease. The incidence of cancer deaths has dropped as a result of significant breakthroughs in cancer treatments and numerous cutting-edge treatment methods (Chowdhury et al., 2016). Surgery, immunotherapy, radiotherapy, stem cell transplant therapy and chemotherapy are among the traditional cancer treatment options (Howell & Valle, 2015). However, there are numerous side effects associated with various therapeutic modalities including mutation, cytotoxicity and multidrug resistance (Tewari et al., 2019). The disease is now being treated with treatments that are frequently invasive, exhibited drug resistance. Apart from treatments, different methods have been used for tumor diagnosis, including x-ray and magnetic resonance imaging (MRI). Although there are many methods for diagnosing cancer, there are still a number of issues including insufficient solubility, fast disabling, poor pharmacokinetics and limited biodiversity that must be resolved before the disease may be correctly and promptly identified (Imran et al., 2019) . However, improved analytical and beneficial horizons have raised the existence frequency of cancer suffered patients, although complete eradication of the illness is still questionable. It is therefore necessary to research and develop new methods for more accurately diagnosing and treating cancer. Nanotheranostics is one such cutting-edge field for the efficient treatment and diagnosis of cancer (Figure 1). Due to their selectivity and tumor homing techniques, nanoparticles have enormous potential in the therapy of cancer (Amir et al., 2022). The side effects of antitumor drugs can be minimized by simply surface fabricating them with cancer-targeting ligands. These have longer in vivo circulation times, which lower injection frequency and boost patient compliance. Nanoparticles are therefore seen as a viable beneficial platform for the treatment of tumor as a result of these benefits. The term ‘Theranostics’, first used by John Funkhouser in 2002, refers to the simultaneous diagnosis and treatment of a disease (Wang et al., 2012). These techniques minimize adverse effects while providing targeted drug delivery to tumor tissues. They also track how the free active entities react with the targeted organ or tissue (Sahoo et al., 2014). Nanotheranostics is known to be created through the combination of nanoparticles with theranostics. Nanotheranostics can be used to diagnose and treat tumor patients in the initial stages (Sohail & Fakhar, 2021). Treatment planning, virtual monitoring of therapy response, and online tracking of therapeutic response are all made possible by multifunctional hybrid nanotheranostics (Anselmo & Mitragotri, 2016). Any nanotheranostic design must consider a variety of factors, including size of the particle, loading capacity, and superficial interactions with the biological environment. For tumor targeting, ideal size from 5 nm to 200 nm of a nanoparticulate system is useful (Lammers et al., 2010).

## 2. Surface functionalization on nanocarriers and targeting strategies

### 2.1. Passive mechanism

Leaky vasculature of the tumor blood vessels enables the nanocarriers to simply enter into the interstitial space by crossing the endothelial barrier. The size of tumor endothelial cell linings varies depending on the type of tumor and ranges from 100 to 700 nm, which is 50–60 times greater than the normal endothelium (Greish, 2007). Moreover, poor lymphatic drainage system in solid tumors results in insufficient circulation to the extravasated cells, leading to the accumulation of the nanocarriers to the tumorous site. This process is referred as enhanced permeability and retention effect (EPR) and it is thought to be a good approach in efficient tumor targeting (Torchilin, 2011). The effective execution of EPR by tumors, as well as tumor characteristics including (pH, angiogenesis and microenvironment) are essential for successful passive targeting (ud Din et al., 2017). Tumor hypoxia physiologically triggers angiogenesis which results in the formation of networks of abnormal blood vessels with enhanced permeation because of large spaces that develop between endothelial cells with a size up to 600 nm (Sibgha et al., 2021). Furthermore, tumor interstitium with reduced lymphatic drainage is also significant. EPR effect has gained notoriety for passive tumor targeting, considering that it is deemed to be the criterion (Hirsjarvi et al., 2011).

**Figure 2.**
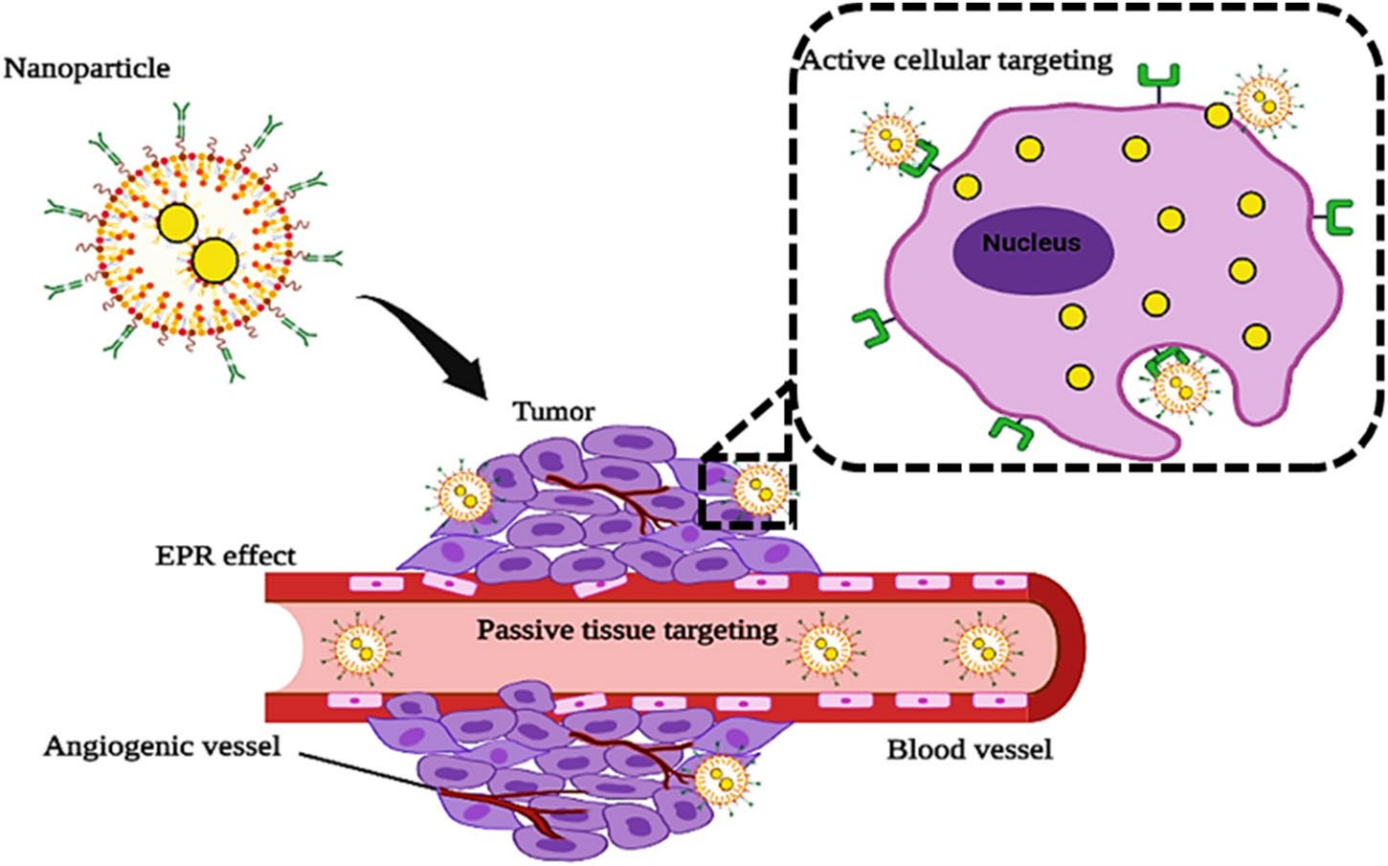
Illustration of the passive and active tumor targeting by nanoparticle (Created with BioRender).

Nanocarriers with low molecular weight drugs reenter into the blood circulation because of diffusion process and are unable to accommodate the tumor site for longer period of time. The pathophysiology and immunochemical conditions of tumor cells completely decide targeting behavior of such drugs, which is known as ‘passive targeting’ (Figure 2). Nanocarriers are not only the source of improvement of the blood circulation of drugs but also enhance the tumor targeting using EPR effect (Haider et al., 2020). To attain the prolonged retention of drugs, a variety of carriers are used, including polymeric and pH-dependent systems. Furthermore, the distinct and dissimilar microenvironment surrounding tumor cells in comparison to normal cells contributes to passive targeting. Rapidly spreading and overactive cancers have an incredibly high metabolic ratio. Due to insufficient oxygen and nutrients, tumor cells obtain additional energy through glycolysis leading to the acidic microenvironment (Orang et al., 2019). Additionally, distinct enzymes like metalloproteases are also released by tumor cells, related to the migration and existence of these cells (Deryugina & Quigley, 2010). Several nanocarriers including liposomes, micelles, polymers, nanoparticles and antibodies were used to target these diverse tumor microenvironments. Different approaches have been used and their potential processes of active and passive targeting to the tumor and endothelium have been described (Ediriwickrema & Saltzman, 2015). Significant advancements have been made in that field, as a considerable percentage of nanocarriers with passive mechanisms of targeting got approved for their medical application. However, the critical shortcomings of passive targeting that can’t be ignored are the misconception of EPR effect, discrepancies among animal models and patients, and limited permeation of the nanocarriers into the desired tissues and tumor cells (Liu & Auguste, 2015).

### 2.2. Active mechanism

Surface modification of the nanocarriers is done with the ligands that bind specifically to their receptors expressed onto the surface of the tumor cells (Goddard et al., 2020). Nanocarriers have large surface area due to their small size and modifiable surfaces that enable several ligands to be conjugated onto their surface, leading to increased specificity (Figure 2). The selection of the ligand is merely dependent on its compatibility within the body, molecular weight, valence and targeting abilities. Commonly used ligands include glycoproteins, growth factors, antibodies, nucleic acids, vitamins and peptides (Sibgha et al., 2021). Active targeting is used to overcome the limitations of passive targeting and get over drug resistance, as well as to minimize the off-target distribution of chemotherapeutic drugs (Kirtane et al., 2013). The specified targeting moiety should selectively conjugate with a receptor that is overexpressed by tumor cells. The particular receptors must also be uniformly expressed in the targeted cells (Anarjan, 2019).

Active targeting is intended to either target the tumorous cells or the tumor microenvironment with nanocarriers decorated with ligands (He et al., 2020). Active targeting of cancer cells is attained by interacting overexpressed receptors with ligand-decorated nanocarriers. Receptor mediated endocytosis enhances the internalization of nanocarriers by tumor cells consequently enhancing drug concentration within the cells. Glycoproteins on cell surface, folate receptors (FR), transferrin receptors (TfRs) and epidermal growth factor receptors (EGFR) are generally targeted overexpressed receptors in different types of tumors (Table 1) (Deshpande et al., 2013; Pérez-Herrero & Fernández-Medarde, 2015). One of these tumor markers may be upregulated on the surface of a respective tumor cells. The impact of docetaxel loaded nanocrystals with transferrin ligands has been evaluated for anticancer activity on A549 cell lines. Ligand conjugated docetaxel nanocrystals have better targeting as compared to unconjugated nanocrystals. Additionally, for improved anticancer activity, cyclic arginyl-glycyl-aspartic acid (RGD) peptide and folate ligand were coated to the surfaces of paclitaxel- and apatinib-containing micelles, respectively (Song et al., 2017). As FR are overexpressed in cancers like breast, ovary, lung and colon thus folate-modified nanocarriers can be used to target these cancerous cells (Guo et al., 2017). Blood-brain barrier endothelial cells have highly expressed TfRs on their surfaces; thus, transferrin as a ligand can be used for the site specific delivery of antitumor drugs into the brain (Gan & Feng, 2014). An alternative approach that actively targets the tumor endothelium rather than the tumor cells has many benefits as compared to the previous one. Attacking the tumor system comprises the devastation of tumor cells, which prevents the tumorous cells from receiving nutrients and oxygen and ultimately leads to the death of the tumors’ cells (Prokopiou et al., 2013). Wu et al determined the antineoplastic effectiveness of paclitaxel-entrapped polymeric micelles in esophageal tumor cell lines (EC9706). In comparison to free drug solution and plain paclitaxel micelles, it was found that intravenous administration of folate anchored paclitaxel-loaded micelles at an equivalent dose to tumor-bearing nude mice resulted in a more effective prevention of tumor growth (Wu et al., 2012). For site specific delivery of antitumor drugs, active targeting is preferred mechanism because it reduces the risk of side effects, improves affinity, increases the amount of drug that reaches the target site and, consequently, boosts the drug’s effectiveness, suppresses multidrug resistance, and has blood-brain barrier crossing potential (Ahmad et al., 2019).

**Table 1.** Brief description of target receptors.

Target receptors	Description	Examples	References
Folate Receptors (FR)	Fundamental component of cell metabolism and DNA synthesis, required by both healthy and tumor cells. Important indicator with increased expression in metastatic cancer cells.	Folate coated micelles with paclitaxel. Folate-iron coated doxorubicin loaded carbon nanotubes	(Li et al., 2011; Wu et al., 2012)
Transferrin Receptors (TfRs)	Iron binding glycoprotein, engaged in cellular development by sustaining iron supply. Overexpressed on the surface of cancer cells.	Lactoferrin and transferrin loaded polymersomes, PEG-PCL loaded polymersomes with transferrin.	(Pang et al., 2011; Nicolas et al., 2013)
CD44 Receptor	Non-kinase glycoprotein with overexpression in liver, breast, cervical and colorectal cancers. Significant marker for cancer stem cells.	Micelles and liposomes conjugated with hyaluronic acid.	(Lin et al., 2017; Lee et al., 2020)
Epidermal growth factor receptors (EGFR)	Transmembrane glycoprotein that activates signal transduction pathways that are involved in regulating proliferation, survival and differentiation of cells.	Cetuximab porphyrin-engrafted carbon dots.	(Wu et al., 2018)

A ‘protein corona’ is created when substantial concentrations of proteins are rapidly coated on nanoparticles, as soon as they enter the blood circulation (Xiao et al., 2021). It can also affect the biodistribution and physicochemical characteristics of the nanoparticles which ultimately influence the binding capacity of ligands to the receptors (Xiao & Gao, 2018). Recently protein corona has gained increased attention and confers nanoparticle a new identity (Farshbaf et al., 2022). Besides this, the protein corona may prevent nanoparticles from specifically targeting tissues and cells (Xu et al., 2022). Salvati et al. reported that the protein corona can prevent the binding of transferrin (Tf) to its cellular receptors and TfR by using Tf coated nanoparticles. Moreover, Wang et al. reported that liposomes coated with folic acid were rapidly engulfed by macrophages due to increased liposomal surface absorption of IgM and with incapability to recognize receptors in vivo. A protein corona, however, can be a key component in directing specific targets (Wang et al., 2020; Xiao et al., 2021).

## 3. Different types of nanotheranostic nanocarriers

**Figure 3.**
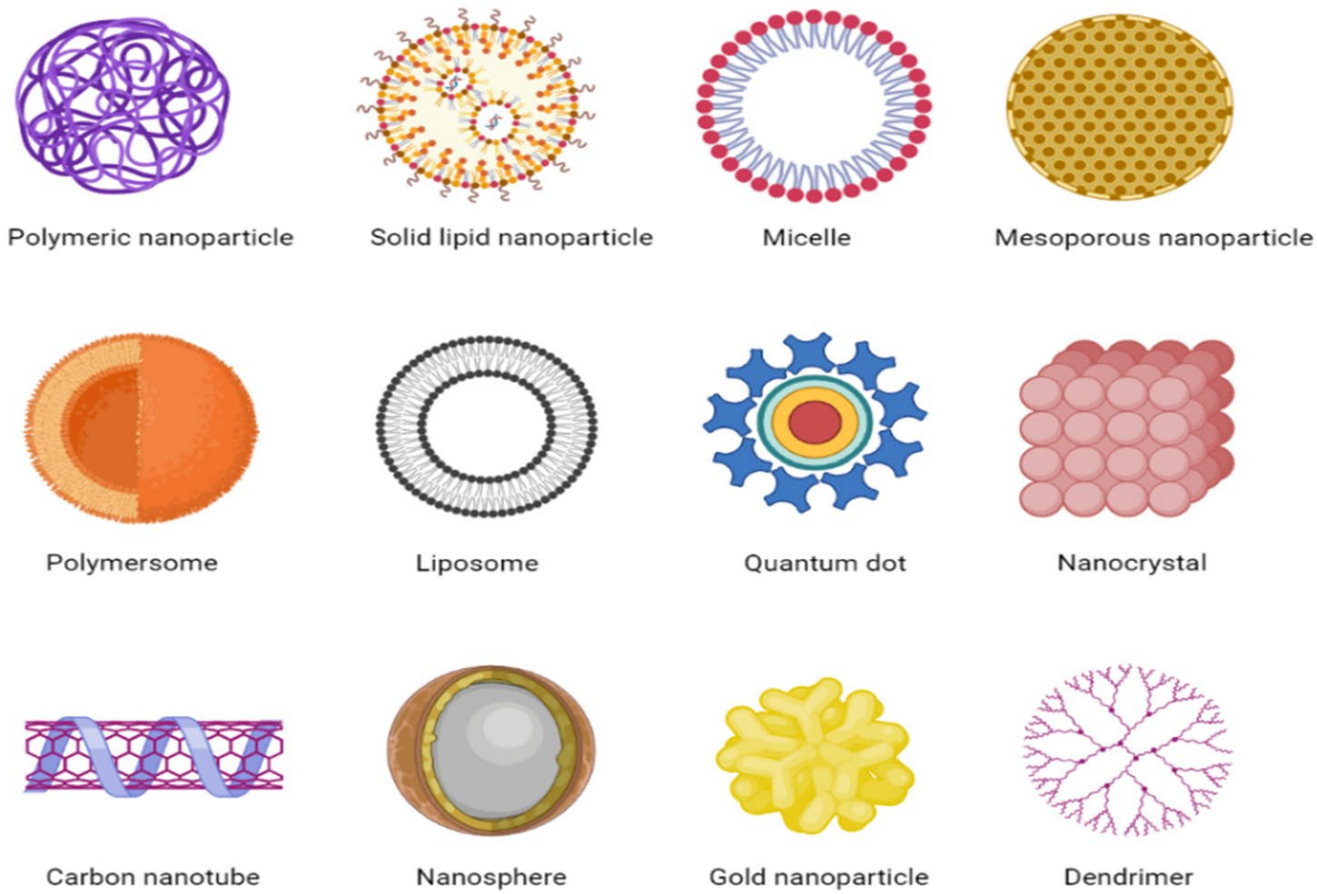
Theranostic nanocarriers used in oncology (Created with BioRender).

Nanocarriers are an essential part of theranostic structures because they serve as a framework for the simultaneous realization of the functions of imaging and therapeutic functions in a single entity (Zeb et al., 2020). Various types of theranostic nanocarriers are listed below (Figure 3).

### 3.1. Nanocarriers based on iron oxide

Significant attention has been given to magnetic nanotheranostics in the field of cancer treatment.

The advantages of magnetic nanocarriers include multimodal imaging, optical imaging, positron emission tomography (PET), MRI, thermal cell apoptosis, improved penetration in cells and also support efficiently gene and drug delivery. Furthermore, magnetic nanocarriers allows the killing of cancer cells by hyperthermia (Shahzad et al., 2021). Local heat generated by magnetic nanoparticle-associated hyperthermia causes the discharge of active moieties that are attached to the magnetic nanocarriers or enclosed in polymeric system (Singh & Sahoo, 2014). These theranostic compounds are up to 100 nm in size, which allows for improved tumor tissue diffusion and enhances distribution (Draz et al., 2014). Magnetic nanocarriers especially iron oxide nanocarriers can be superficial conjugated to antitumor drugs, targeted agents, and biodegradable polymers to decrease their cytotoxicity (Xie et al., 2011). The system was composed of terbium incorporated PEG coupled with GdPO_4_ nanorice altered with cerium and glutamic acid and other components to enhance the multifunctional properties of iron oxide nanoparticles. The iron oxide nanoparticle-containing biphasic system displayed green light glowing properties with effective water stability. This was filled with the antitumor medication doxorubicin and used cell lines including HeLa and MCF-7 to demonstrate cell death in vitro. It was believed that this multimodal system was a potent chemo-thermal tumor treatment and imaging vehicle (Sahu et al., 2014). This nanocarrier system was exploited for site specific activity in breast and colon tumor cell lines using superparamagnetic iron oxide nanocarriers coupled with poly (styrene)-b-poly (acrylic acid), folic acid and drug doxorubicin (Patra et al., 2014).

### 3.2. Nanocarriers based on gold and silver

Other than iron, a variety of gold and silver nanoparticles can be effortlessly created with different superficial alterations, making them extra friendly and minute cytotoxic (Boisselier & Astruc, 2009). The advantages of gold/silver nanocarriers include easy to manufacture, multimodal imaging and cell apoptosis by photothermal therapy and photodynamic therapy (Dixit et al., 2015). To specifically inhibit the HeLa cells, gold nanoclusters entrapped with the suicide gene CD-UPRT (cytosine deaminase-uracil phosphoribosyltransferase) and 5-fuorocytosine remained utilized (Sahoo et al., 2014). Gold nanostructures can specifically and photothermally target urothelial tumor cells when coupled with anti-mucin 7 antibodies (Chen et al., 2015a). In order to improve site specific therapy for the breast tumor by photothermal treatment, a nanotheranostic system made of gold nanocarriers aptamer and graphene oxide were developed. This platform had no impact on normal cells, even at low doses (L. Yang et al., 2015). This nanoplatform has benefits like increased biocompatibility, site specificity and cancer cell death. The MUC1-conjugated aptamer interacted with the glycoprotein (MUC1) to target breast tumor cells using the self-centered aptamer attached gold nanocarrier graphene oxide nanoparticulate system. The near-infrared light caused AptAuNP-GO to temporarily increase the expression of HSP70, which thereafter decreased and led to permanent cell demise. Combined use of the heat and a HSP70 inhibitor lead to the apoptosis of breast cancer cells. Therefore, this could result in the creation of these inhibitors entrapped Apt-AuNP-GO that could produce heat to the breast tumor tissues, additional enhancing healing capabilities with reduce side effects. With the use of high contrast imaging equipment, gold-core or gold-shell nanocarriers linked with specific probes demonstrated effective targeted activity with help of PTT in A549 cells (H. Shi et al., 2014). Lung tumor, melanoma, and breast tumor tissues have all shown anticancer activity when exposed to biosynthesized silver nanoparticles, which glow brilliant red inside cells (S. Mukherjee et al., 2014). Another research scientist designed gold nanocluster AuNC@CBSAICG@HA composed of red emission bovine serum albumin entrapped indocyanine green and later conjugated with hyaluronic acid (HA) and the reported nanoplatform provided effective photothermal treatment for breast tumor (R. Liu et al., 2019). Another research scientist fabricated furin-responsive gold nanocarriers delivery system (AuNPs-D&H-CABT) with RK peptide (RVRRCK)-AuNPs (AuNPs-D&H-RK) and modified with 2-cyano-6-amino-benzothiazole-polyethylene (CABT), and later conjugated with drug doxorubicin (DOX) and hydroxychloroquine (HCQ). This nanoplatform system improved the breast tumor targeting and simultaneously overcome drug resistance (Xie et al., 2021). Another research group of scientists designed nanoplatform (AuNPs-A&C) composed of Ala-Ala-AsnCys-Lys altered AuNPs (AuNPs-AK) and 2-cyano-6-aminobenzothiazole modified AuNPs (AuNPs-CABT) and later conjugated with DOX. This reported nanoplatform provided effective nanoparticle tumor accumulation and the potential to improve therapeutic outcome in brain cancer (S. Ruan et al., 2016). Another group of scientists fabricated gold nanoparticles composed of chemotherapy and immunotherapy. The designed nanoplatform legumain-responsive AuNPs (D&H-AA&C) along with anti-PD-L1 antibody could further improved the antiglioma effect and effectively prevent recurrence (Ruan et al., 2019).

### 3.3. Nanocarriers based on protein

Therapeutic and diagnostic methods have been carried out using protein-based nanotheranostic substances. The advantages of nanocarriers based on protein include, it allows both interior and exterior surface alteration and the ferritin nanocages have affinity for human transferrin receptor-1 naturally (Z. Wang et al., 2016). Due to their proteinaceous nature, manufactured nanocages can be created on both their interior and exterior sites. According to research ferritin nanocarriers have also been utilized as effective tumor nanotheranostics. This is because the ferritin heavy (H)-chain has a high attraction toward human transferrin receptor-1 (CD71), that is upregulated in cancer cells (Truffi et al., 2016). Nanoparticles made of lipoprotein have been employed as theranostic tumor treatments (K. K. Ng et al., 2011). A multifunctional cancer-associated site specific delivery system and two-modal imaging-assisted co-loaded therapy for tumor have both been created using nanotheranostics based on albumin. To enable magnetic resonance imaging, human albumin along with a photosensitizer entity chlorine e6 (Ce6) that simultaneously assisted moiety for Mn^2+^. The cancer-specific peptide Arg-Gly-Asp (cRGDyK) was then employed to stimulate the self-gathering of Ce6 changed HAS coupled with the anticancer medication paclitaxel, which targets αvβ3-integrin upregulated on cancer cells. Co-delivery of HSA-Ce6 and HSA-RGD as well as the formation of a HSARGD-HSA-Ce6 core-shell assembly resulted in the development of two different types of nanostructures. These structures allowed for both chemo-photodynamic treatment, and when HSA-Ce6-PTXRGD-managed cells were subjected to irradiation by light, synergistic cancer cell death was seen. Tumor specific targeting of RGD attached nanoparticles after systemic injection was demonstrated by two-way imaging in vivo. After intravenous administration, the HSA- Ce6- PTX- RGD-1 nanoparticulate system demonstrated combined photodynamic and chemotherapy. Additionally, compared to separate monotherapies, the combination of phototherapy and chemotherapy was more successful (Q. Chen et al., 2015b). The ability to function as possible cancer theranostic agents has been proved by semiconductor nanocrystals made with the assistance of a protein-based nano reactor (T. Yang et al., 2016).

### 3.4. Nanocarriers based on silica

Porous silica nanocarriers linked to the iRGD peptide were utilized as nanotheranostic modalities. The advantages include increase porosity and image-guided drug delivery (C.F. Wang et al., 2015). In order to treat the pancreatic tumor cells, silica nano clamps were combined with topotecan, daunorubicin, and quantum dots as possible nanotheranostic agents (Muhammad et al., 2014). According to the authors Chan & Lin (2015) mesoporous silica nanoparticles attached to lanthanide ions like gadolinium and europium were utilized as effective theranostic entities to choose tumor cells while concurrently with imaging (MRI and fluorescent). Additional study found that mesoporous silica nanocarriers could prevent breast tumors that overexpress CD44, leading to photothermal ablation, when coupled through hyaluronic acid and pegylated lipid-loaded silica and carbon nanocrystals (Q. He et al., 2012). When combined with hematoporphyrin and docetaxel as part of trifunctional treatment and a bifunctional imaging vehicle, these mesoporous silica nanocarriers were investigated as possible nanotheranostic entities (Q. Chen et al., 2015b).

### 3.5. Nanocarriers based on lipid

Lipid-based drug delivery systems have received the most attention (G. Yu et al., 2021). The advantages include easy to manufacture, extended period of circulation, increase specificity and reduce toxicity (Draz et al., 2014). Liposomes are spherical, self-assembling nanocarriers with lipid bilayer walls around an aqueous core, they can contain both hydrophilic and hydrophobic active moieties. The primary type of nanocarrier drug delivery system to be used for medicinal uses was liposomes, which are still the focus of current research (Jamshaid & Ud Din, 2021). Drug administration with liposomes is highly advantageous due to their improved therapeutic index, biocompatibility and biodegradability (Amoabediny et al., 2018). Doxil® was the first nanotherapeutic medicine that the FDA used for tumor treatment, because it is a doxorubicin (Dox)-encapsulating PEGylated liposome (Danhier et al., 2010). Although non-targeted nanocarriers like Doxil® first appeared to have good outcomes, their uses have been constrained by a number of issues, the most significant of which is their nonspecific targeting (G.X. Liu et al., 2014). Many studies have described the manufacture of bi-targeting liposomes with improved advantages and superior beneficial results in order to address this problem. Yuan et al. (2016) designed liposomes for the double delivery of antitumor medications doxorubicin and paclitaxel, for the therapy of skin cancer using peptide (TAT) and transferrin (Tf). The (HIV) type-1 trans-activator protein TAT, is essential for virus replication. TAT peptide has two lysine’s and six arginine residues. They interact more easily with the negatively charged plasma membrane because of their cationic charges, which increases the plasma membrane permeability. TAT might therefore arrive cells when it combines with huge or small scale compounds and transport them effectively by both receptor and transporter independent and unsaturated channels to the targeted cells (Kluza et al., 2012). While DOX interferes with DNA by preventing macromolecular formation, PTX prevents the normal breakdown of microtubules. Using flow cytometry, the in vitro uptake of cell research of B16 cells were examined. According to the findings, dual-modified liposomes showed more cellular absorption than unaltered, Tf-altered, and TAT-altered liposomes, by 14-, 8.7-, and 2.8-fold, respectively. Additionally, apoptosis experiments revealed that liposomes bi-conjugated by Tf and TAT had an increase level of necrotic and apoptotic result than un-altered, Tf-altered and TAT-altered liposomes (Yuan et al., 2016). A dual-site specific nanocarrier made of PTX-entrapped liposomes and the plasmid that contains green shining protein (EGFP), is altered by folic acid and hyaluronic acid (HA) was described by G.X. Liu et al. (2014). Positively charged FA-modified liposomes cooperate with complexes and combine to form masses. To stop that FA-modified liposomes was covered with HA, a negatively part of the extracellular matrix. HA covered liposomes linked to CD44 that is abundantly stated in different kinds of tumor cells. In addition, an enzyme would degrade the HA coating on the liposomes surface, exposing the FA entity and directing it toward the tumor cells. The in vitro cytotoxicity of these liposomes was examined using the MTT test by using two different cell lines (hepatocellular carcinoma cell line (HepG2) and murine malignant melanoma cell line (B16). Drug-free HA/FA/liposome treatment increased the cell viability of both cells more than drug-free FA/liposome treatment did. Additionally, HA/FA/liposomes demonstrated higher cellular absorption values at 0.5 h compared to FA/liposomes, demonstrating that HA showed biocompatible coating and increasing the values of internalization (G.X. Liu et al., 2014).

### 3.6. Nanocarriers based on polymer

In the past ten years, the usage of biodegradable and biocompatible polymers in drug delivery has expanded quickly (Yousaf et al., 2021). Numerous research studies have discussed the function of pharmacological entities that can be conjugated to the polymer to extend their half-lives and improve their targeting (Manandhar et al., 2021). The advantages include gene and drug delivery therapy and controlled-release or stimuli-induced -release of drugs (W. Xu et al., 2022). In one study, HUVECs and HeLa cells were treated with RGD and Tf-functionalized poly [(amine-ester)-co-(D, L-lactide)]/1,2-dipalmitoyl-sn-glycero-3-phosphoethanolamine copolymer (HPAE-co-PLA/DPPE) nanoparticles. Active targeting of the cancer cells resulted from the co-loading of the nanocarriers with Tf and RGD. RGD improved site specific delivery, passage, and nanoparticle accumulation to tumor areas by conveying integrin, and Tf improved the cell uptake of nanocarriers in tumor tissues that express TfR. RGD raised the cytotoxic amount in HUVECs overexpressing the α5β3 integrin by ten times, while Tf boosted the cytotoxicity in HeLa cells overexpressing the Tf receptor by two times. For cells of HUVECs, the IC_50_ of RGD- and bi-modified nano particulate systems was lesser than that of unmodified, Tf-altered, and free-PTX nanoparticles. Though, for HeLa cells, the IC_50_ of bi-modified and Tf-conjugated nanocarriers decreased in alternative to free-PTX, non-altered, and RGD-altered nanocarriers. Given that HUVECs convey a great number of α5β3 integrin receptors but few TfR, this discovery makes sense given that RGD-modified nanoparticles would be more hazardous to cells. On the other hand, HeLa cells, whose surface is coated with Tf receptors responded better to Tf-modified nanoparticles. Additionally bi-modified nanocarriers displayed 1.3 and 1.8 times increased cellular uptake by HUVECs and HeLa cells as contrast to unaltered nanocarriers correspondingly (Q. Xu et al., 2012). In their study, Sun et al. (2017) they created nanocarriers that target brain tumor cells by combining a tumor homing peptide (AP1) with DOX-entrapped polylactic acid (PLA) nanocarriers. AP1 linked to the IL-4R that is anticipated on the brain tumor. AP1-co-loaded nanoparticles demonstrated greater cellular absorption that was also correlated with the concentration of nanoparticles. In C6 cells, AP1-altered carriers were approximately twice as likely to be taken up as unaltered particles. Furthermore, the IC_50_ for any formulation of DOX in C6 cells was 194.3 ng/mL for free DOX, 48.68 ng/mL for AP1-altered carriers, and 112.8 ng/mL for unaltered carriers. In the animal investigations, those mice treated with AP1-loaded carriers survived longer (47 days) than mice with original carriers (35 days), additional demonstrating the effectiveness of targeting approaches (Sun et al., 2017).

### 3.7. Nanocarriers based on carbon

Carbon nanotubes and graphene oxide nanocarriers are the most prevalent carbon-based nanocarriers. Due to their distinct physical and chemical characteristics, research into using carbon nanotubes (CNT) as drug distribution entities has undoubtedly accelerated recently. The advantages of graphene nanocarriers include increase surface area, improve colloidal stability, image-guided photothermal activity, optical absorbance and super-paramagnetism (Shi et al., 2013). Due to the extraordinarily high surface area of CNTs, the nanotube wall can accommodate significant therapeutic loading. Additionally, the polyaromatic surface of CNTs makes it simple to bind supramolecular aromatic compounds like DOX. In order to cure brain glioma, carbon nanotubes (OMWNT) linked with angiopeptide-2 sequence (OMWNT/ANG) was developed. After administering the carriers O-MWNT/ANG’s to glioma-bearing mice, the fluorescence image of the animals was examined to check the ability to target gliomas in vivo. The results revealed that OMWNT/ANG did collect in glioma in a significantly increase manner then OMWNT did strengthen the targeting in glioma cells (Ren et al., 2012). In a related study, a bi-targeting delivery system for DOX into HeLa cells was created using folic acid and iron-assisted carbon nanotubes (FA-MWCNT-Fe). The FA-MWCNT-Fe detected HeLa cells through a site specific method and attached to the cells through passive targeting as a result of being linked by folate and the iron entities, concurrently. The usage of magnetic field provides 1–3 times enhancement of the cytotoxic effects induced by FA-MWCNT-Fe as contrast to without iron carriers (R. Li et al., 2011). For drug and gene delivery graphene has been utilized, since its discovery in 2004 due to its capacity to cross the cellular membrane and enhance the cell uptake of many compounds. Additionally, important, graphene has a very high surface area due to all its atoms are uncovered on its surface, which significantly improves the binding and loading of different kinds of moieties. Graphene is frequently converted to graphene oxide (GO) to add functional groups containing oxygen to increase the carrier’s hydrophilicity. Due to the abundance of hydrophilic groups, including hydroxyl, epoxide, and carboxylic groups, graphene oxide (GO) is readily diffused in water-based environments, which is one of its key advantages. Additionally, GO is a promising medium for drug and gene delivery systems owing to its significant biocompatibility (Xiong et al., 2010). The adsorption of GO and DOX has also been shown to be pH-sensitive, providing a low release when mixing in blood and a full release into the endosomal pH after cellular internalization. Numerous studies in recent years have conclusively shown that GO can destroy tumor cells in vitro and reduce size of the tumor in vivo when exposed to NIR. In order to treat CT-26 cells with chemo-phototherapy, GO nanoparticles that was pH-sensitive and site specific targeting was created. Due to the abundance of EGFR on the surface of colon tumor cell lines, it was altered using PEG and a specific monoclonal antibody against EGFR (cetuximab). The IC_50_ value of altered-GO entities against CT-26 cells was lowered than non-altered vehicles, according to in vitro cytotoxicity data. More interestingly, after using photothermal therapy with NIR laser light, the IC_50_ value was additional decreased to 1.17 μg/mL. Results of in vivo antitumor studies showed that at day 14 in BALB/c mice, the relative tumor volume was 22.6 times lower then mice treated with DOX-entrapped GO nanoparticulate system and 13.8 times lesser then mice with magnetic DOX entrapped GO nanoparticles (lacking phototherapy) (Y.J. Lu et al., 2018).

### 3.8. Nanocarriers based on distinctive lipid

Porphysomes could be employed to optically visualize targeted antitumor PTT, PDT- and PTT-mediated antitumor properties, multimodal imaging and photoacoustic imaging for diagnosis and measuring its biodistribution (Tang et al., 2018). The porphysomes, which are nanocarriers that mimic porphyrinlipid hybrid liposomes, were created by a research team from China and Canada. Through PDT and PTT, this nanosystem was discovered to be beneficial for the targeted destruction of tumor cells. Additionally, to enhance the tumor targeting, these spherical nanostructures made of pyro-lipids can be specially modified and coupled with certain ligands (Jin et al., 2014). Pyro-lipids modified by apolipoprotein-E have also been developed as a target for U87 glioblastoma cells. Tumor cells were examined utilizing NIR fluorescence imaging, and it was discovered that they contain more nanocarriers than normal cells do. Additionally, it was discovered that the site specific PTT and PDT treatment was linked to a roughly 80% decrease in viable cancer cells (Rajora et al., 2017). In actuality, porphysomes are multimodal imaging structures as related to those seen in inorganic nanoparticles. These have the capacity to eliminate primary tumors as well as remove metastasized lymph nodes without causing damage to nearby tissues (Muhanna et al., 2015). Radionuclide tagged ^64^Cu-porphysomes have been developed and tested against orthotropic prostate and bone metastatic tumors using an all-in-one approach. Through the use of PET and fluorescence imaging, the outcomes demonstrated targeted tumor killing (T.W. Liu et al., 2013).

### 3.9. Nanocarriers based on virus

Virus nanoparticles (VNPs) also known as virus-like particles (VLPs), are self-assembled, resilient protein cages that are roughly 100 nm in size and have homogeneous nanostructures (Xiong et al., 2010). VNPs (viruses as nanocontainers) have newly received wide attention for use in nanotechnology applications such as drug delivery, diagnosing, gene therapy and targeted delivery (Pattenden et al., 2005). VNPs have been studied for use in nanotechnology and drug delivery. These viruses come from a variety of sources, including plants tobacco mosaic virus and cowpea mottle virus, bacteria (Qβ, MS2) and animals (adenovirus) (Obraztsov et al., 2007). VNPs, an emerging platform for nanocarriers, have a number of appealing qualities, such as morphological regularity, biocompatibility, simplicity in surface modification, and accessibility in a range of sizes and shapes (Ma et al., 2012). VNPs are able to happen the demands of drug nanoparticles, including biocompatibility, hydrophilicity, and improved drug loading skill, thanks to the flexibility of chemical and genetic alterations that may be made to their surface. VNPs’ circulation period within the host can also be increased by PEGylating the surface of the particles (Y.J. Lu et al., 2018). Drugs can either be chemically or physically bonded to the surface of VNPs for drug delivery applications (Douglas & Young, 2006). By using some viruses’ natural affinity for overexpressed receptors in different tumors or by chemically or genetically altering the surface of VNPs. VNPs can be used as drug-carrying nanocontainers to target particular cancer targets. In several investigations, the tumor targeting potential of VNPs loaded with chemotherapeutic drugs has been examined (P. Singh et al., 2007).

## 4. Nanoparticles in clinical translation

Within 0.08 seconds, a google scholar search for ‘nanoformulations, cancer treatment’ produced about 3000 results; more than 18,000 papers are based on nanomedicines. However, it is disappointing to see that a lot of this fundamental research was unable to be applied in clinical settings. There are still only a few nanomedicines that have been approved to treat cancer, 25 years after the first nanochemodrug, Doxil, was released onto the market in 1995. Only a relatively small number of formulations have progressed into the clinical stage over time, despite the enormous number of research attempts to employ nanoparticles for cancer therapeutic purposes. There aren’t many nano-formulations that have received the green light to enter the market as cancer treatments, according to a cursory glance at the NP-based formulations currently in clinical studies and on the market (Mundekkad & Cho, 2022). While there are currently up to 75 nanoformulations undergoing clinical trials, the FDA has only approved 16 nano-based cancer medicines (He et al., 2019). Table 2 enlist the nanoparticles that are currently in clinical trials for treatment of oncology. Table 3 enlist the nanoparticles that are clinically approved for treatment of oncology.

**Table 2.** Nanocarrier-based drug delivery systems that are under clinical trials for cancer therapy.

Nanocarrier	Generic name	Formulation	Active ingredient	Phase, indication and clinical trial identifier number	References
AGuIX nanocarriers	*	Combination of gadolinium nanocarriers with chemoradiation and brachytherapy	Polysiloxane Gadolinium	III, Advanced cervical tumor & brain metastases, NCT03308604	(Bilynsky et al., 2022)
Polymeric nanocarriers	CRLX101	Pegylated based cyclodextrin nanocarriers	Camptothecin	II, prostate cancer, NCT02187302	(Egusquiaguirre et al., 2012)
Polymeric micelle	NC-6004	Pegylated based polyglutamic acid micelle	Cisplatin	I/II, non-small cell lung tumor, NCT02240238	(Q. Zhou et al., 2018)
Polymer-drug conjugated nanocarriers	ProLindac™	Diaminocyclohexane platinum polymer prodrug	Oxaliplatin	II, ovarian tumor	(Nowotnik & Cvitkovic, 2009)
Liposomes	Thermodox™	Pegylated liposomes activated by heat	Doxorubicin	II, Breast tumor, NCT02536183	(Chaudhry et al., 2022)
Gold nanocarriers	AuroShell	(PEG-coated silica gold nanoshells)	*	II, Prostate tumor, NCT04240639	(Rastinehad et al., 2019)
Silica nanocarriers	Cornell Dots (C -Dots)	PEGylated and exterior surface covered with tumor targeting peptide (cRGDY)	Near-infrared fuorophore and ^124^I	I, Image-assisted intraoperative mapping of nodal metastases, NCT02106598	(C. Anselmo & Mitragotri 2019)
Carbon nanocarriers	*	Intraoperative injection of carbon nanocarriers	*	Not applicable, Colorectal cancer, NCT03350945	(Y. Zhang et al., 2019)
Iron oxide nanocarriers (SPIONS)	Ferumoxytol	Polyglucose sorbitol carboxy methyl ether covered SPIONs	*	I, Head and neck tumor, NCT01895829	(Madamsetty et al., 2019)

**Table 3.** Clinically accepted nanoformulations for cancer treatment.

S.No	Trade name along with company	Type of nanocarrier with particle size/Targeting mechanism	Indication	Year approved	Route of administration	References
1	Doxil (Janssen)	Liposomal doxorubicin (PEGylated), 80–90 nm, passive targeting	Ovarian carcinoma, AIDS-related Kaposi’s sarcoma	FDA in 1995 EMA in 1996	Intravenous injection	(Anselmo & Mitragotri, 2019)
2	Zinostatin stimalamer (Pharma of Yamanouchi and Astellas)	Copolymer conjugated formulation of Zinostatin with polystyrene-co-maleic acid-half-butylate, passive targeting	Hepatocellular carcinoma (HCC)	Japan in 1994	Intra-arterial injection	(T.W. Liu et al., 2013)
3	Genexol (Samyang Biopharm)	Paclitaxel loaded polymeric micelle, 20–50 nm, passive targeting	Breast cancer that is metastatic and recurrent	South Korea in 2006	Intravenous injection	(Weissig et al., 2014)
4	Eligard (Tolmar)	Polymeric matrix product of leuprolide acetate, passive targeting	Prostate cancer	FDA in 2002	Subcutaneous injection	(Werner et al., 2013)
5	DepoCyt (SkyPharma Inc.)	Liposomal cytarabine, 10–20 µm, passive targeting	Lymphomatous meningitis	FDA in 2007	Intrathecal injection	(Thakor & Gambhir, 2013)
6	Oncaspar (Enzon Pharma)	Covalent coloaded formulation of PEG with L-asparaginase, 50–200 nm, passive targeting	Acute lymphocytic leukemia	FDA in 1994	Intravenous or intramuscular injection	(Dinndorf et al., 2007)
7	Kadcyla (Roche)	Conjugation of herceptin to microtubule assembly inhibitor, active targeting	Early-stage HER2-positive breast tumor	FDA in 2019	Intravenous injection	(Alphandery et al., 2015)
8	NanoTherm (MagForce)	Superparamagnetic iron coated with aminosilane (hyperthermia, treatment), 20 nm, magnetic targeting	Glioblastoma	EMA in 2013	Intratumoral injection	(Martinelli et al., 2019)

## 5. Effect of nanocarrier shape on tumor deposition and therapeutic efficacy

Size, shape, charge, and surface coating of NPs are physicochemical characteristics that affect both tissue biodistribution and tumor uptake (Zein et al., 2020). When a nanoparticle’s shape is altered, the way ligands are presented eventually has an impact on how readily they connect to other nanoparticles. It is anticipated that a nanoparticle shape will impact the pace of tumor deposition and therapeutic effectiveness since it influences a nanoparticle blood circulation, ability to marginate and binding affinity. Table 4 provides the impact of various nanocarriers shapes on tumor penetration (Toy et al., 2014). Table 5 summarizes various strategies, advantages and limitations of targeted nanocarriers.

**Table 4.** Effect of various nanoparticle shapes on tumor penetration.

Nanocarrier type	Nanocarrier shape	Treatment	Therapeutic outcome	References
Gold nanocarriers	Nanohydrogel, spherical NPs, cylindrical, nanorods	3D spheroid model	Improved outcome of cylindrical shaped hydrogel nanocarriers	(Agarwal et al., 2015)
Micelles	Filamentous, spherical	Mice xenograft tumor	Increased tumor accumulation of filamentous	(Christian et al., 2009)
Single-walled carbon nanotube (SWNT)	Carbon nanotubes	Tumor in mouse	Tumor targeting	(Z. Liu et al., 2007)
Gold nanocarriers	Spherical, rod, hollow	Human endothelial cell uptake	Increased cellular uptake for spherical form as compared to hollow shapes	(Bartczak et al., 2012)
Gold nanocarriers	Nanorods, nanospheres	Photothermal triggered therapy	10 fold increased photothermal absorption efficiency as compared to nanospheres	(Barua et al., 2013)
Silver nanoparticles (AgNPs)	Spherical, triangular, nanorods	Skin permeability in hairless mouse	Maximum penetration showed by nanorods	(Tak et al., 2015)
Non spherical polystyrene particles	Spherical, filamentous	Tumor	Improved tumor homing showed by spherical forms	(Champion & Mitragotri, 2009)
Non-cross-linked polystyrene (PS)	Spheres, ellipsoids, rectangular disks	Uptake by macrophages (phagocytosis)	Negligible phagocytosis showed by elongated nanocarriers	(Champion & Mitragotri, 2009)
Antibody conjugated nanocarriers	Nanorods, nanospheres	BT-74 breast tumor cells	i. 5 fold increased cellular uptake as compared to nanospheres ii. 66% increased binding and cellular uptake as compared to nanospheres	(Barua et al., 2013)
Gold nanocarriers	Nanorods, spheroids, nanoshells, hollow nanospheres	Shallow skin tumor and deeper tumor	i. Nanospheres for shallow tumor ii. Nanospheres and nanorods for deep tumor	(Kessentini & Barchiesi, 2012)
PEGylated tobacco mosaic virus	Nanorods, nanospheres	Blood circulation	Extended circulation of nanorods as compared to nanospheres	(Bruckman et al., 2014)
Paclitaxel-entrapped filomicelles	Spherical, filamentous (filomicelles)	Blood vessels of rats and mouse	Extended circulation of filomicelles	(Geng et al., 2007)
Iron oxide nanoparticles (α_v_β_3_ integrin-targeted nanochain)	Nanochains, spherical form	Orthotopic 4T1 mammary adenocarcinoma in mouse	i-Two fold increased tumor targeting then their spherical form ii-40% increased localization in primary tumor	(Peiris et al., 2012)

**Table 5.** Summarizes various strategies, advantages and limitations of targeted nanocarriers.

Nanocarrier	Therapeutic agent	Cancer	Targeting strategy	Advantages/targeting efficiency	Limitations	References
Liposome	Doxorubicin (Dox)	Metastatic breast cancer	Passive	Half-life increased by 100 fold, reduce systemic toxicity	i-EPR effect is not possible in all tumors, ii-chances of drug expulsion and multiple drug resistance	(Gabizon & Martin, 1997)
Liposome	Oxaliplatin	Pancreatic tumor	Active targeting (transferin receptor targeted)	Showed high targeting and delivery efficacy, averting nonspecific binding and the MDR efflux mechanism	i-Ligand-mediated targeting approaches have not yet made an important clinical influence on human health	(Yu et al., 2012)
Iron oxide nanocarriers	MRI contrast agent	Glioblastoma multiforme (GBM)	Antibody based targeting (EGFR receptor)	Increase selectivity and binding affinity for the interest area	i-Large size and trouble in conjugation to nanocarriers ii-expensive to manufacture, iii. potentially induce an immunogenic response	(Gao et al., 2004)
Supramagnetic iron oxide nanoparticles (SPIONS)	siRNA	Breast cancer	Peptide based targeting (EPPT peptide)	Small size, reduce immunogenicity, ease of manufacture	i-Reduce target affinity ii-vulnerability to proteolytic cleavage	(Perey et al., 1992)
Gold nanospheres	Irinotecan	Cervical cancer	Small molecule based targeting (Folate targeting)	Low cost to produce, targeted delivery of both imaging and therapeutic moities to tumor sites	Decrease circulation time	(Lu et al., 2010)
Iron oxide nanocarriers	Doxorubicin	Prostate cancer	Aptamer based targeting (PSMA aptamer)	Bind to targeted area with increase specificity, advantage over antibodies small size (15 kDa), low immunogenicity easy to manufacture	Fast blood clearance due to degradation of nuclease	(Yu et al., 2011)

## 6. Role of nanocarriers in specific tumor targeting

Mutations in either the genes regulating cellular proliferation and differentiation or the protein sequence influencing cell inhibitory action and apoptosis are the primary causes of tumors. These defective genes cause the development of tumorous cells, which have the exclusive properties of abnormal cell growth, the incapability to stop unnecessary cell division, inhibition of apoptotic cell death, and the capacity to infiltrate nearby and far-off tissues (Fulbright et al., 2017). Radiation, chemical agents, hereditary factors, and some viruses are potential causes for genetic changes (Moses et al., 2018).

Conventional therapies for the treatment of tumors are associated with many side effects including cardiac, renal, GI tract and hepatic toxicities. Researchers are developing site specific drug delivery systems to minimize the off-target effects of antitumor drugs, thereby enhancing therapeutic efficacy of the cytotoxic agents (Aravind et al., 2012). Nanocarriers and their applications in several types of tumors have been explained due to their occurrence and high death rates.

### 6.1. Breast tumor

Doxorubicin, paclitaxel, and cisplatin are just a few of the chemotherapeutic drugs that are commonly used for treating breast tumors in both neoadjuvant and adjuvant therapies (Khan et al., 2022). The first-line treatment for breast tumors is doxorubicin. By blocking DNA and macromolecular synthesis within tumor cells, it slows the proliferation of tumor cells. Alopecia, increase in neutrophil count, and heart issues are the three main side effects of doxorubicin that are linked to its toxicity with increasing doses. Breast tumors are typically treated with drugs like cisplatin or oxaliplatin, in combination with other anticancer drugs. By maintaining DNA binding and cross-linking, cisplatin causes apoptotic cell death and stops the proliferation of tumorous cells. Most notable adverse effects include neurotoxicity, ototoxicity and nephrotoxicity, associated to higher plasma concentrations (Schmitt & Page, 2018). In order to improve the safety of combination drugs for the treating cancers, a variety of antitumor drug-loaded nanocarriers were used as new techniques for the site-specific drug delivery. For the treatment of estrogen receptor-positive breast tumors, biocompatible poly caprolactone nanocarriers loaded with tamoxifen were synthesized. This study claimed that by administering drug directly to the estrogen receptor, the formulations of selective estrogen receptors, including tamoxifen, might improve their therapeutic efficacy (Maji et al., 2014; Mamnoon et al., 2021). Moreover, a pH-sensitive delivery system using poly-ethylene oxide (PEO)-altered poly-amino ester nanocarriers were established for the administration of paclitaxel as an anticancer drug for breast tumors. In comparison to other synthetic polyesters, the PEO-Poly-amino ester nanocarriers had a rapid degradation profile and were in the nanosized range (Shahin & Lavasanifar, 2010). By utilizing synthetic peptide ligands, P18-4, Shahin et al. had created doxorubicin (DOX) loaded liposomes to target breast cancer. By altering the quantity of P18-4, it was possible to examine the impact of the ligand on breast cancer in terms of cytotoxicity and growth arrest. It was observed that choosing optimum density can increase the anticancer activity of the modified P18-4 peptide (Shahin et al., 2013).

Multidrug resistance (MDR) is the main cause of unresponsive cancer behavior and its reoccurrence. To achieve this, Milane et al created paclitaxel and lonidamine-loaded EGFR-targeted polymeric nanocarriers for the treatment of breast tumor. Their findings showed that these nanocarriers with properties such as effective drug encapsulation and controlled drug release led to better combination therapy with efficient EGFR targeting. The efficient utilization of co-delivery systems based on nanocarriers is an emerging strategy for the treatment of various tumors. Such methods have not only solved some challenges but have also resulted in enhanced therapeutic effects with reduced cytotoxicity when administered in prolonged and targeted drug delivery forms (Shahin et al., 2013).

### 6.2. Lung tumor

For several decades, lung cancer remains the foremost cause of cancer related deaths with 154,050 estimated deaths in 2018 worldwide. It is most common prevalent malignancy found in men (Bray et al., 2018; Siegel et al., 2019). Inefficiency of available therapies and late diagnosis contribute to overall poor survival rate. Furthermore, the tumor metastatic to secondary locations is responsible for high mortality rates (Muthoosamy et al., 2016). Survival rate in lung tumors mainly rely on early diagnosis and the most preferred approach is surgical excision. The tumor cells are routinely becoming resistant to drugs, so the available therapeutic mediators are faced with poor outcomes and low survival rates, i.e. only <20% per five-year. Among available lung cancer therapies, the most widely used treatment strategy is chemotherapy. The major impediment which retards the clinical success of chemotherapeutic agents is their inadequate concentration at tumor site. And to address this challenge, high concentration of drugs are being repeatedly used which ultimately lead to toxic effects (A. Mukherjee et al., 2019). Other issues associated with existing chemotherapy are its poor site specificity and low treatment efficiency. So, to attain adequate therapeutic outcomes regarding lung cancer, there is a dire need to develop site specific treatment variables.Recent advancements in theranostics nanomedicine has served as a propitious scheme in cancer treatment. Conventional diagnostic methods were inappropriate choices for cancer screening as they were faced with problems like expensive procedures and inaccuracy (Aggarwal et al., 2014). To meet this challenge, cheap and noninvasive sensor based gold nanoparticles were developed successfully and were utilized in diagnosing lung cancers (Peng et al., 2009; Ishtiaq et al., 2020). Similarly, doxorubicin loaded in poly (butyl cyanoacrylate) nanoparticles were found effective in lung tumor (Roa et al., 2011). Moreover, 9-nitrocamptothecin loaded liposomal formulation have shown efficacy against advanced lung tumors both in in-vitro and in-vivo settings (Verschraegen et al., 2004). The incompetency of antineoplastic agents to demolish cancerous cells could be well compensated by oncolytic viruses-based gene therapies (Beljanski & Hiscott, 2012). Likewise, cowpea mosaic virus (CPMV) having an average size of ∼27 nm exhibits a great potential to be used in vaccination therapy for lung tumors (Robertson et al., 2011).

### 6.3. Pancreatic tumor

Inspite of a very low incidence rate (approximately 3%), pancreatic tumor is still regarded as fourth major cause of cancer deaths among both genders in United States. The prevalence of pancreatic tumor is higher (50%) in men than in women. It usually affects adults, with cases arising in 60–80 years old patients (R. Hruban, 2010). Pancreatic tumor is associated with very low survival rates, i.e. 5–7% per 5 years (Adiseshaiah et al., 2016) . Average survival rates are 6–10 months and 3–6 months for localized pancreatic tumor and metastatic pancreatic tumor respectively. Several factors are responsible for low survival rate, mainly the end stage diagnosis. Moreover, most of the patients did not show any symptom until the disease progress to a metastatic stage. About 10% of the patients suffering from pancreatic cancer are eligible for initial resection (Gillen et al., 2010). Numerous risk factors for pancreatic tumor have been identified such as familial basis (R.H. Hruban et al., 2010), smoking (Iodice et al., 2008), pancreatitis (Raimondi et al., 2010), and diabetes mellitus (Bosetti et al., 2014), but currently there is no tool for screening patients with greater risk.

Treatment options for pancreatic tumor includes surgical excision, chemotherapy and radiation which are used in multidisciplinary way on the basis of stage of tumor. Among available schemes, chemotherapy is most widely utilized in treating the metastatic pancreatic adenocarcinoma, with gemcitabine serving as frontline therapeutic agent (Y-J. Li et al., 2020). FLORINAX, a novel chemotherapeutic regimen showed improvement in survival rate when compared to gemcitabine-based therapy. However, according to a recent study, both these agents were unable to improve therapeutic outcome while treating metastatic pancreatic tumor in clinical trials (Conroy et al., 2018).

Recently, nanotechnology has shown enormous applications in the field of cancer including improvements in tumor diagnostics, imaging and treating or preventing diseases via site specific delivery (Grodzinski et al., 2019). Similarly, siRNA loaded liposomes were used against HER-2 in pancreatic tumor (X. Liu et al., 2011). Albumin coated paclitaxel nanocarriers, were also used to treat pancreatic tumors. Additionally, iron oxide nanoparticles were synthesized in order to express a surface-targeted uPAR moiety.

### 6.4. Colorectal tumor

Colorectal cancer manifests as malignant neoplasm in colon and/or rectal mucosa, which is currently fourth most widely diagnosed cancer globally (Torre et al., 2015; Cisterna et al., 2016). Environmental factors are usually responsible for genetic mutation, whereas geographical factors cause colorectal cancer in various populations of world. Colon cancer occurs due to mucosal colonic polyps, which are further classified into two histological types named as adenomatous and hyperplastic polyps. Similarly, hyperplastic polyps are composed of reduced cytoplasmic mucus but enhanced number of glandular cells (Lawrance et al., 2006). Probability to develop a colon cancer increases with the increase in number of adenomas polyps, which gets worse in case of familial adenomatous polyps or if colectomy is not performed.

Currently, there are various treatment approaches which are of potential use against colorectal cancer, i.e. cryosurgery, radiation, chemotherapy and surgery. Among these, chemotherapy is preferred mostly owing to improved life quality and patient compliance associated with it (Chuah et al., 2013). In recent times, various types of drug loaded nanoparticles, i.e. polymeric nanoparticles, liposomes, micelles and dendrimers were developed in nanosize range (20–400 nm) that strongly impacted drug delivery for chemotherapy (Xing et al., 2021; Raja et al., 2022). However, the main limitation faced with chemotherapy is the small amount of drug available to the tumor area, rendering it less efficient option. Recently, the advancements in nanotechnology discipline have led to development of several nanocarriers to achieve desired outcomes regarding the treatment of colorectal cancer. Such nanocarriers were used for approved delivery of antitumor drugs including capecitabine, irinotecan, bevacizumab, 5-FU and oxaliplatin (Din et al., 2017a; Din et al., 2017b). For the treatment of colorectal cancer, current liposomal formulations under clinical study that has completed Phase II clinical trials are CPX-1, LE-SN38 and Thermodox; CPX-1 (Tolcher & Mayer, 2018). Similarly, polymeric nanocarriers serves as solid applicants for drug delivery in cancer therapy owing to their potential of encapsulating both hydrophilic and hydrophobic drugs (Kamaly et al., 2016). Another successful approach of cancer therapy in colorectal cancer is through targeting tumor cells via conjugation of ligands (antibodies, aptamers, small molecules and peptides) on the surface of nanoparticles. These ligands are incorporated by chemical modification during the synthesis of nanoparticles (Shahin et al., 2011).

## 7. Role of nanomedicine in conventional tumor therapy

Use of nanomedicine in the therapy is known as nanotherapeutics (nanocarrier based therapeutics) (Qiao et al., 2019). Nanotherapeutics have been identified as promising alternatives to many of the risks raised by the free drugs. A contemporary use of nanotherapeutics has a significant impact on the medical industry. The development of nanotherapeutics opens up new possibilities for enhancing the efficacy and safety of conventional medicines (Hunt et al., 2022).The advancements in nanotherapeutics have enhanced the applications in conventional treatments, i.e. through photothermal, photodynamic and gene therapies.

### 7.1. Photothermal therapy (PTT)

**Figure 4.**
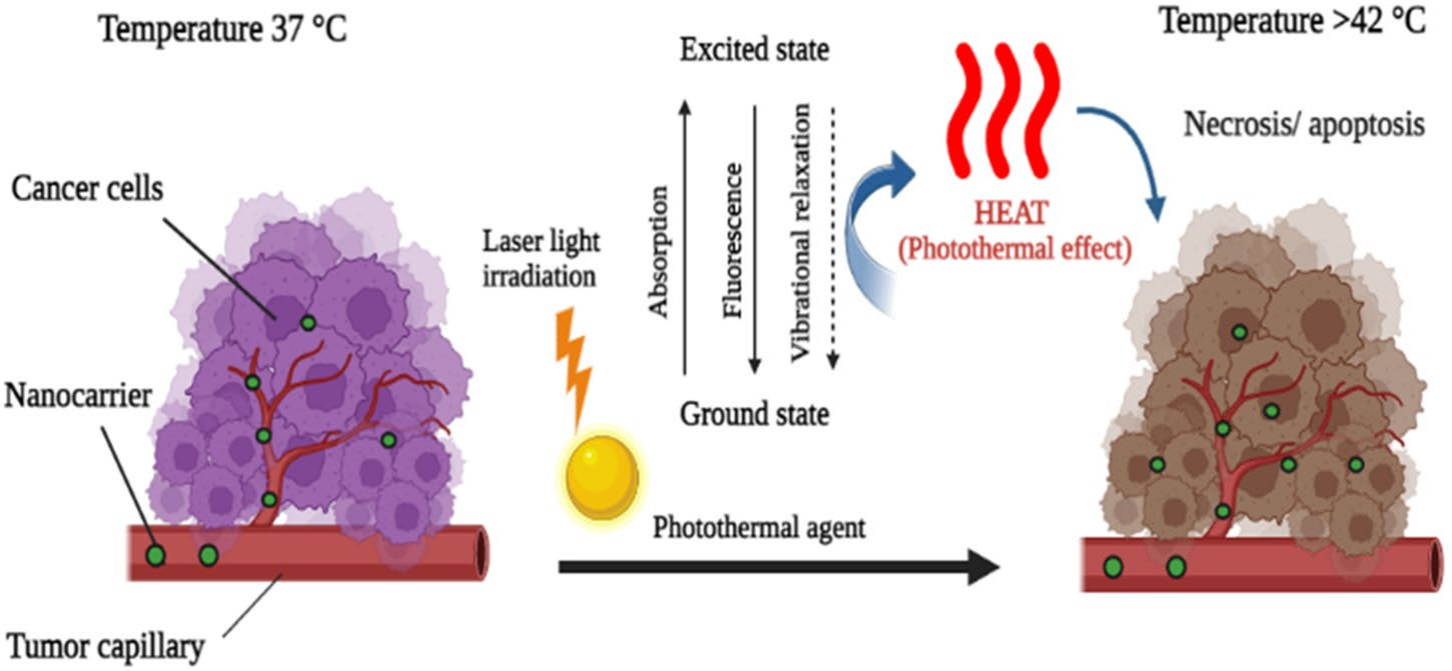
Illustration of photothermal cancer therapy (Created with BioRender).

An effective cancer treatment known as photothermal therapy uses photothermal materials to precisely warm the cancer’s target site, to thermally decompose it (Montaseri et al., 2020). Photoactive compounds are given to patients during photothermal therapy; after being exposed to radiation at a target site, the photoactive become excited, converting the energy to heat while coming back to ground state (Figure 4). The ensuing hyperthermia can result in permanent cell damage at 42–46 °C over the course of 10 minutes for tissues that do not receive enough blood and oxygen. The greater the temperature provided; less exposure time is required. By boosting blood flow and tumor vascular permeability, this strategy has been successfully employed to eradicate tumors or improve the effectiveness of drug delivery (Doughty et al., 2019; G. Gao et al., 2021). Steel nanoparticles, metallic nanostructures and chromophores like indocyanine green, naphthalocyanine, and porphyrin coupled with transition metals, are examples of these photothermal agents. Electromagnetic energy, such as microwaves and radio waves, damage cells by denaturing proteins and membranes during the thermal treatment of malignancies. Due to the high molecular density in water, iron oxide nanoparticles are commonly used photothermal agents with controllable absorption potential. Iron oxide nanoparticles suspended in water have been thought to provide heat when injected directly into the tumor site (Zhongling Wang et al., 2017).

### 7.2. Photodynamic therapy (PDT)

**Figure 5.**
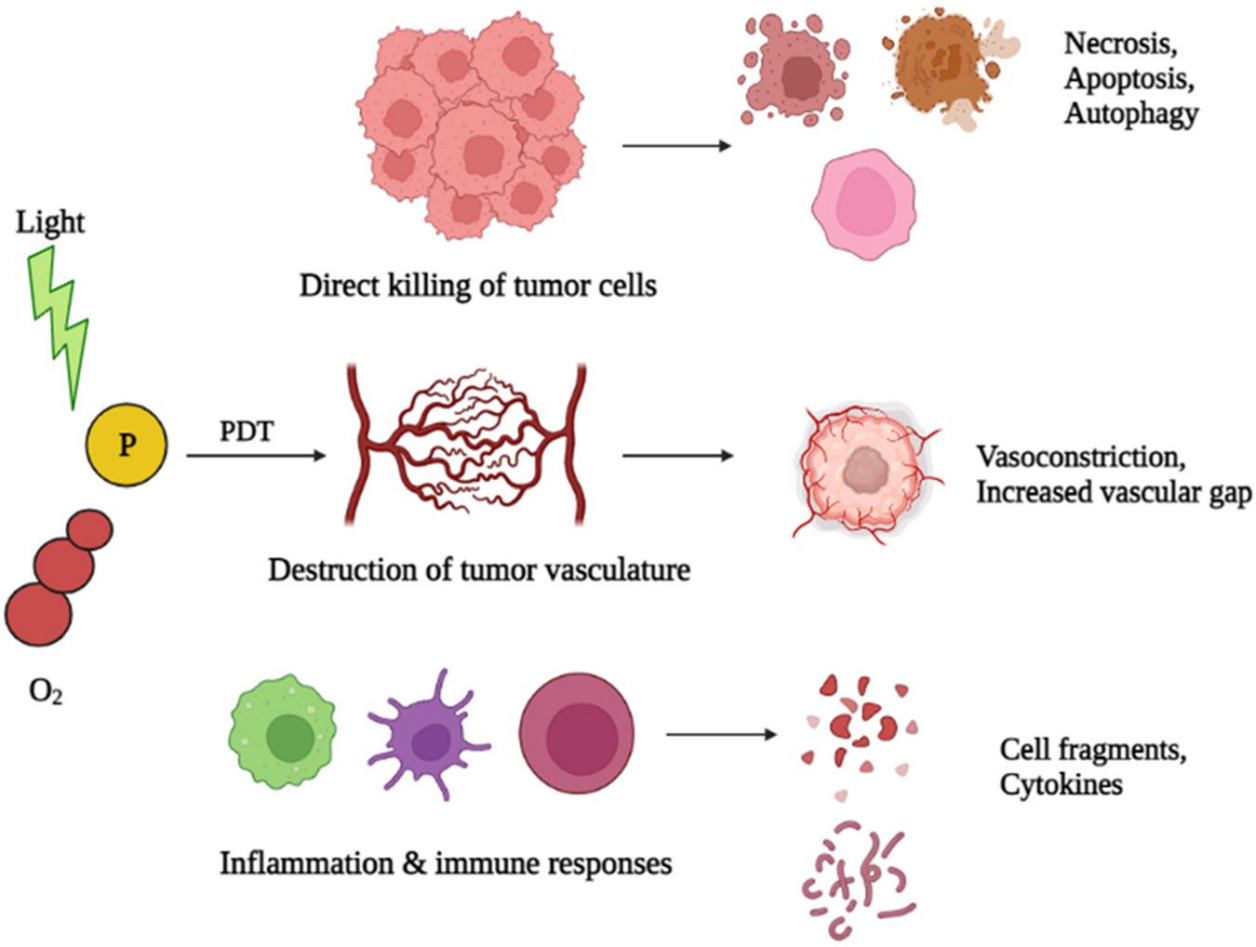
Mechanisms of photodynamic therapy (Created with BioRender).

PDT involves the utilization of photosensitizers for cancer treatment (Figure 5). Photosensitizers distribute the light to the nearby media when exposed to the appropriate laser light, creating oxygen radicals that trigger apoptosis (Allison et al., 2010). PDT has many benefits over PTT, including the fact that it needs lesser intensity of light to provide the beneficial impact than PTT, that needs light with a high-power density. Additionally, it has fewer side effects, has minimal toxicity, and improves patients’ wellbeing. Clinical studies have shown that PDT is beneficial in treating a variety of tumors, including bladder, lung, esophageal, and oral tumors. FDA has approved some photosensitizers for the treatment of cancer, including Laserphyrin, Photofrin, Metvix, Visudyne, Foscan, Levulan and Hexvix (Chilakamarthi & Giribabu, 2017).

To improve the PDT effect, Zhang et al. developed a gold cube nanocomposite loaded with doxorubicin having coating of mesoporous silica (X. Zhang et al., 2019). Wan et al. developed a nanocarrier system for co delivery of indocyanine green and doxorubicin containing ammonium bicarbonate and oxyhemoglobin. PDT effectiveness is enhanced via using oxyhemoglobin (Wan et al., 2018). Two-photon PDT, which uses two photons to facilitate energy absorption at lower energy NIR region, is also investigated in PDT. This method provides improved penetration and precise targeting in tumor cells (Ogawa & Kobuke, 2008).

Numerous studies have demonstrated that the therapy with combination of chemotherapy and PDT, also known as chemo-photodynamic therapy, can make tumor cells more vulnerable to chemotherapeutics, and have a more effective synergistic antitumor impact (C. Lin et al., 2020). PDT is another preferable strategy like other anticancer drugs (such as doxorubicin, oxaliplatin, cyclophosphamide, and the like) for inducing immunogenic cell death (ICD). It might enhance the release of antigens from the tumor cells, triggering the process of downstream immunological regulation against the tumor, which would then encourage the development of dendritic cells and T lymphocytes activation. Therefore, it is considered that the utilization of PDT and MET together will increase the effectiveness of antitumor immunity (Hu et al., 2021). Moreover, Yang et al. developed morpholine based bilirubin nanoparticles, containing diclofenac and a photosensitizer, chlorin e6 to overcome resistance caused by hypoxia (Y. Zhou et al., 2022). Wenfeng et al. described a novel nanocarrier system having chlorin e6, berberrubine, metalloproteinase peptide forming a triblock structure coated with PEG-histidine with shape changing ability, charge reversal, chemo photodynamic effect and increased circulation in blood (Jia et al., 2022). Similarly, I-P@NPs@M macrophage membrane coated shape adjustable nanoparticles were prepared for breast cancer therapy with increased blood circulation, tumor site specificity, drug release and efficient chemotherapy (R. Liu et al., 2020). Furthermore, (C/I)BP@B-A(D)&M1m coated with phagocytes having the ability to change size, control drug release and laser responsiveness, were developed for increased tumor targeting and effectiveness (Hu et al., 2020).

### 7.3. Gene therapy

Gene therapy in the treatment of cancer plays a significant role. In this procedures, the genetic material is delivered intravenously (Sabir et al., 2019). However, because nucleic acids are susceptible to nucleases breakdown and rapid clearance in blood circulation, a vector is required for protection and delivering the genomic material to the target site. This treatment offers a great asset for disease treatment by controlling outflow of tumor and activating the genes which deliver healing proteins. On the basis of this, a variety of approaches have been developed to date such as RNA silencing, miRNA-based and self-destructive gene therapy using a transgene that prevents tumorous growth after being presented to tumor cells. Genes and sRNAs can be loaded to nanocarriers by van der Waals interaction or by conjugation to the surface of nanocarriers. Inorganic nanocarriers, polymeric nanocarriers and all other carriers for the treatment of malignant growth genes have all been widely used in research on novel cancer treatments. A PEI-based hybrid polymer nanoparticles were prepared containing hyaluronic acid and PEG forming a polymeric system by mixing with small interfering RNA (siRNA) (Mattheolabakis et al., 2016). Additionally, inorganic nanocarriers like carbon based nanotubes, gold nanocarriers, quantum dots, and others have been used in the gene therapy for cancer. Oishi and coworkers prepared gold nanocarriers with siRNA incorporated in it and introduced these nanocarriers to the HuH7 liver cancer cell lines to determine the efficacy (Oishi et al., 2006).

## 8. Future perspective

The development of new-generation nanocarrier medications is one of the major obstacles to current advancements in nanotechnology being employed effectively for the therapy of many cancers. By interacting with the receptors on the chosen cells and tissues as well as the surface-attached ligand, this expansion would validate the energetic targeting of malignancies. But there are challenges to be resolved, including absence of adequate knowledge, struggle in piercing the cell membrane, a limited therapeutic window for medications, regulatory challenges, and cost-effectiveness. Nanoparticles have the ability to reach the site specific set for cancer therapies, both in traditional and next-generation drugs. Regrettably, the typical reappearance of formulation-driven expansion has not obtained the expected compliance of individual. In numerous tumor-modeling animals, various targeted nanocarriers have demonstrated increased therapeutic effectiveness. More specifically, there are over 120 clinical trials in progress involving numerous formulations that comprises nanocarriers for antibodies. Similar to this, scientists can now visualize the nature and location of the tumor, which helps them to paradigmatize the best treatment plans. Furthermore, a vehicle with increased half-life in circulation and increased capacity for targeting surface antigen is desired, if the cancer cells are of the circulating types like lymphoma and leukemia. Additionally, it is predicted that scientists will soon be able to create site specific molecular composites that could result in improved therapeutic outcomes with reduced costs. Only, a rare of these promising preclinical drug delivery systems have made it to market, despite the fact that researchers have explored a good quantity of innovative drug delivery methods to increase treatment efficacy in patients. Despite the encouraging outcomes of pre-clinical research, it is essential for academia and industry to work together on research to support additional investigations and the advancement of promising nanotheranostics candidates into clinical trials. These nanomaterials are multifunctional agents due to their capacity to combine different cargoes, and in the future, a better thoughtful of the interactions among their physicochemical characteristics and the biological microenvironment on their in vivo study is required to strengthen their clinical translation. To get around these issues, it is crucial to change some of the established models. In this regard, exceptionally multifarious efforts are needed to quickly fix a few problems in order to attain the safe use of the prepared nanoparticles in clinical research. These involve the creation of typical nanoformulations that have had their efficacy, safety, and possible toxicities tested both in vitro and in vivo studies. There are numerous nano-based cancer treatments available on the market. However, in order to ensure a secure and efficient medication administration for the treatment of cancer, the aforementioned applications require a thorough clinical study. Additionally, personalized therapies can be designed based on each patient’s unique molecular and genetic profile.

## 9. Conclusion

Nanomedicine is one of the most rapidly growing approach for treating cancer. Numerous nanocarriers have been discussed that can be used for therapy of different tumors with enhanced permeation and therapeutic effects of antitumor drugs. The hope of treating tumors has been boosted by these advancements in cancer treatment and the remarkable creation of various novel drug delivery systems. Future drug dosage management is anticipated to increase the usage of nanocarrier systems for the administration of antitumor drugs while simultaneously minimizing side effects and maximizing systemic drug release from the nanocarriers.
